# Supplementary material for: Modeling glioblastoma heterogeneity as a dynamic network of cell states
Source: Mol Syst Biol. 2021 Sep 16;17(9):e10105. doi: 10.15252/msb.202010105 (PMC8444284; doi:10.15252/msb.202010105)
Supplement: Supplementary file 5 — Source Data for Figure 3 [file MSB-17-e10105-s001.zip › Figure3A_sourcedata/GSEA_3065/hallmarks_state2.GseaPreranked.1623416269049/gsea_report_for_na_pos_1623416269049.html]

Report for na\_pos 1623416269049 [GSEA]

| GS  follow link to MSigDB | GS DETAILS | SIZE | ES | NES | NOM p-val | FDR q-val | FWER p-val | RANK AT MAX | LEADING EDGE || 1 | HALLMARK\_E2F\_TARGETS | Details ... | 196 | 0.82 | 2.95 | 0.000 | 0.000 | 0.000 | 1147 | tags=75%, list=12%, signal=84% |
| 2 | HALLMARK\_G2M\_CHECKPOINT | Details ... | 191 | 0.80 | 2.85 | 0.000 | 0.000 | 0.000 | 1004 | tags=58%, list=11%, signal=63% |
| 3 | HALLMARK\_MYC\_TARGETS\_V1 | Details ... | 194 | 0.78 | 2.80 | 0.000 | 0.000 | 0.000 | 1589 | tags=78%, list=17%, signal=92% |
| 4 | HALLMARK\_DNA\_REPAIR | Details ... | 131 | 0.62 | 2.13 | 0.000 | 0.000 | 0.000 | 1148 | tags=33%, list=12%, signal=37% |
| 5 | HALLMARK\_OXIDATIVE\_PHOSPHORYLATION | Details ... | 191 | 0.59 | 2.11 | 0.000 | 0.000 | 0.000 | 2284 | tags=56%, list=24%, signal=72% |
| 6 | HALLMARK\_MYC\_TARGETS\_V2 | Details ... | 51 | 0.67 | 2.02 | 0.000 | 0.000 | 0.000 | 1583 | tags=45%, list=17%, signal=54% |
| 7 | HALLMARK\_SPERMATOGENESIS | Details ... | 58 | 0.64 | 1.99 | 0.000 | 0.000 | 0.000 | 954 | tags=33%, list=10%, signal=36% |
| 8 | HALLMARK\_MITOTIC\_SPINDLE | Details ... | 183 | 0.53 | 1.90 | 0.000 | 0.000 | 0.000 | 912 | tags=26%, list=10%, signal=28% |
| 9 | HALLMARK\_UNFOLDED\_PROTEIN\_RESPONSE | Details ... | 97 | 0.39 | 1.30 | 0.083 | 0.169 | 0.961 | 1104 | tags=27%, list=12%, signal=30% |
| 10 | HALLMARK\_MTORC1\_SIGNALING | Details ... | 179 | 0.35 | 1.26 | 0.072 | 0.200 | 0.982 | 1793 | tags=37%, list=19%, signal=45% |
| 11 | HALLMARK\_PI3K\_AKT\_MTOR\_SIGNALING | Details ... | 83 | 0.38 | 1.24 | 0.138 | 0.207 | 0.991 | 1093 | tags=20%, list=12%, signal=23% |
| 12 | HALLMARK\_FATTY\_ACID\_METABOLISM | Details ... | 108 | 0.37 | 1.24 | 0.132 | 0.198 | 0.994 | 3045 | tags=50%, list=32%, signal=73% |
| 13 | HALLMARK\_PEROXISOME | Details ... | 67 | 0.39 | 1.23 | 0.143 | 0.195 | 0.997 | 1951 | tags=31%, list=21%, signal=39% |
| 14 | HALLMARK\_REACTIVE\_OXYGEN\_SPECIES\_PATHWAY | Details ... | 38 | 0.38 | 1.09 | 0.335 | 0.399 | 1.000 | 1763 | tags=32%, list=19%, signal=39% |
| 15 | HALLMARK\_UV\_RESPONSE\_UP | Details ... | 113 | 0.31 | 1.03 | 0.411 | 0.497 | 1.000 | 1508 | tags=27%, list=16%, signal=31% |
| 16 | HALLMARK\_ESTROGEN\_RESPONSE\_LATE | Details ... | 113 | 0.30 | 1.01 | 0.447 | 0.500 | 1.000 | 1602 | tags=25%, list=17%, signal=29% |
| 17 | HALLMARK\_ALLOGRAFT\_REJECTION | Details ... | 60 | 0.31 | 0.97 | 0.489 | 0.542 | 1.000 | 1538 | tags=30%, list=16%, signal=36% |
| 18 | HALLMARK\_ADIPOGENESIS | Details ... | 156 | 0.22 | 0.76 | 0.921 | 0.885 | 1.000 | 2355 | tags=33%, list=25%, signal=43% |
Table: Gene sets enriched in phenotype **na **[plain text format]****
